# Epidemiology and survival of patients with central nervous system solitary fibrous tumors: A population-based analysis

**DOI:** 10.3389/fonc.2022.977629

**Published:** 2023-01-23

**Authors:** Taikun Lu, Haiyang Xu, Xuechao Dong, Zheng Jin, Yubo Wang

**Affiliations:** Department of Neurosurgery, First Hospital of Jilin University, Changchun, Jilin, China

**Keywords:** primary brain tumor, solitary fibrous tumors, hemangiopericytoma, SEER program, CNS disease

## Abstract

**Background:**

The objective of this study was to determine population-based estimates of the epidemiology and prognosis of central nervous system solitary fibrous tumors (cSFTs).

**Methods:**

We extracted the data of patients diagnosed with cSFTs between 2004 and 2018 from the Surveillance, Epidemiology, and End Results database. We analyzed the distribution of patients according to their demographic and clinical characteristics. Binary logistic regression analysis was performed to predict which patients would be diagnosed with malignant cSFT. Possible prognostic indicators were analyzed by multivariable Cox proportional hazards models.

**Results:**

A total of 650 cases were included. The majority of patients were diagnosed at 50-59 years old, and the median age at diagnosis was 55 years. A total of 13.4% of the tumors were located in the spinal canal, and 24% of the tumors were benign. Most of the tumors were larger than 3 cm, but distant metastasis was rare. Tumor resection was the first choice of treatment for these patients, and total resection was achieved in 51.1%. Radiation therapy after surgery was also administered to 42.3% of the patients. The median survival was 57 months. Intracranial tumors and tumors with distant metastasis tended to be malignant. The results of the log-rank test showed that the patients who underwent total resection had better overall survival (OS), but the effect of radiation therapy after surgery was not significant.

**Conclusion:**

cSFT is a rare and aggressive type of tumor. Tumor resection is the first choice for treatment, and radiation therapy after surgery does not improve OS. Patients older than 60 years of age who are diagnosed with intracranial tumors, malignant tumors and distant metastasis have worse OS outcomes than their counterparts.

## Introduction

Central nervous system solitary fibrous tumors (cSFTs), also known as solitary fibrous tumor/hemangiopericytoma, are very rare, accounting for only < 1% of all primary central nervous system (CNS) tumors (1). Hemangiopericytomas were first described by Stout and Murray in 1942 (2, 3), and the first cSFT was reported by Carneiro in 1996 (4, 5). There is a large overlap in the histopathological and clinical characteristics of these two tumor types (4), and recent genetic research has shown that the majority of SFTs and HPCs have inversions at chromosome 12q13 and exhibit NAB2-STAT6 fusion (6). In the 2016 WHO classification of tumors of the CNS, they were integrated into a new entity, “solitary fibrous tumor/hemangiopericytoma (SFT/HPC)” (1, 4), defined as a mesenchymal tumor of fibroblastic type often showing a rich branching vascular pattern, encompassing a histological spectrum of tumors previously classified separately as meningeal SFTs and hemangiopericytomas (1). In the 2021 WHO classification of tumors of the CNS, the term hemangiopericytoma was retired, with SFTs replacing the terms SFT/HPC (7). In the statistical report published by the Central Brain Tumor Registry of the United States (CBTRUS), because of their rarity, cSFTs are grouped with other mesenchymal tumors of the meninges (8, 9). Because of the rarity of SFT, we found that the epidemiology and prognosis of patients with primary cSFTs have not been well documented and thus remain unclear. The Surveillance, Epidemiology, and End Results (SEER) program of the National Cancer Institute covers approximately 48.0 percent of the U.S. population (10). Therefore, we conducted this population-based cohort analysis to explore the clinical features and prognosis of patients with cSFT.

## Method

### Data extraction from the SEER database

The term solitary fibrous tumor was defined according to the third edition of the International Classification of Diseases for Oncology (ICD-O-3), including codes 8815 and 9150. The research period was set from 2004 to 2018. Patients of an unknown age were excluded. Detailed patient data were obtained by case listing sessions using SEER 18 Regs Custom Data (November 2018 submission) (11). All the data were obtained with the SEER*Stat 8.3.9 program. Data on the age at diagnosis, sex, race, tumor size, tumor location, pathology type, presence of distant metastasis, treatment and follow-up were extracted. The surgery codes were interpreted according to the 2022 SEER Program Coding and Staging Manual (12). Because the data from SEER are publicly available for research purposes, ethics committee approval and informed consent were not necessary to perform the analyses.

### Statistical analysis

Descriptive analyses were conducted to evaluate the distribution and tumor-related characteristics of patients with cSFTs. Binary logistic regression analysis was performed to predict which patients would be diagnosed with malignant tumors (8815/3 and 9150/3). The Kaplan–Meier method was used to estimate overall survival (OS), and the intergroup differences were assessed using log‐rank tests. Survival was defined as the time from diagnosis to the date of death from any cause. Possible prognostic indicators were analyzed by the multivariable Cox proportional hazards models. Statistical significance was defined as p < 0.05. All the data were analyzed by IBM SPSS Statistics 25 software (IBM Corporation, Armonk, New York, USA).

## Results

**Figure 1:**
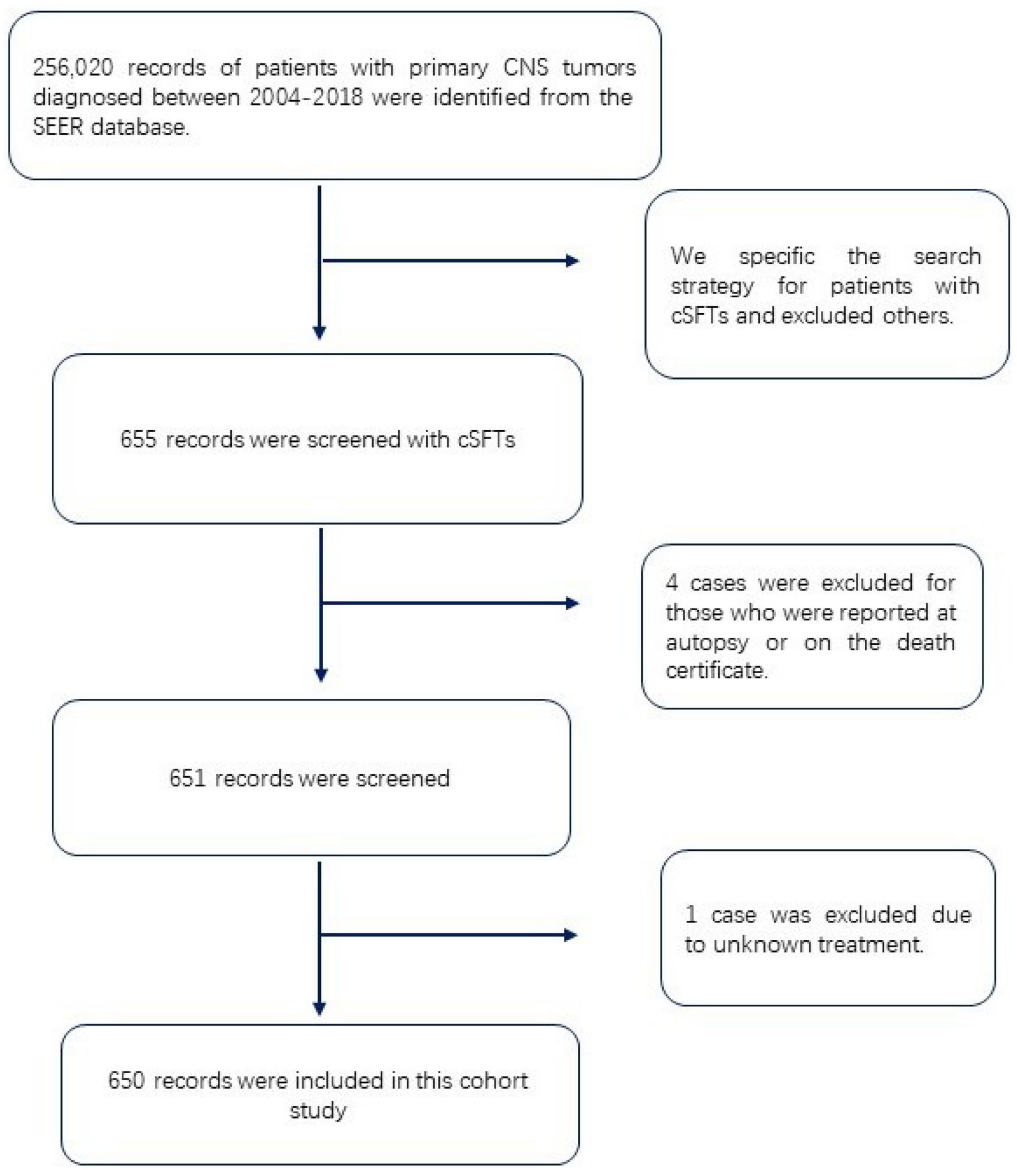
Flow diagram of the study.

A total of 655 cases of cSFTs were indexed from 256,020 cases of primary CNS tumors, accounting for only 0.26% of all primary CNS tumors. We excluded four cases that were reported at autopsy or on the death certificate, and we excluded one case due to unknown treatment. Finally, 650 cases were included. The flowchart was presented in **Figure 1**.

**Figure 2:**
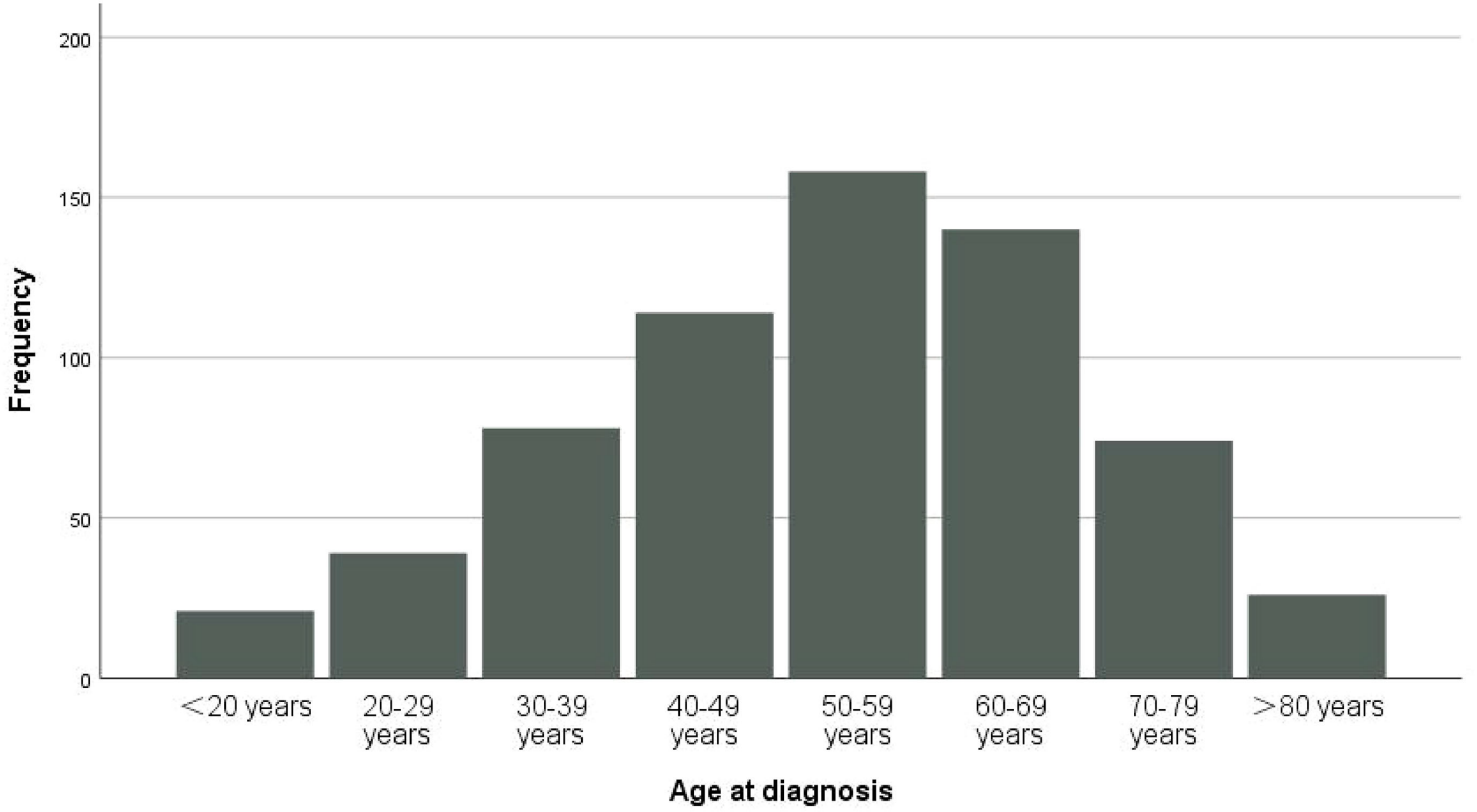
The distribution of the patients based on age at diagnosis.

The demographic characteristics, treatment and follow-up information for these patients are presented in **Table 1**. There were 322 females and 328 males with a median age at diagnosis of 55 years. The majority of patients were diagnosed at 50-59 years old (**Figure 2**). Most of the patients were non-Hispanic (n=530, 81.5%). A total of 80.6% of the tumors were intracranial (n=524), and 13.4% of the tumors were located in the spinal canal. Benign tumors (8815/0 and 9150/0) accounted for only 24% of the total (n=156) in this cohort. In addition, 61.5% of the tumors were larger than 3 cm at diagnosis (n=400). Distant metastasis was very rare, accounting for only 3.1% (n=20), and the lung was the most frequent location of metastases. A total of 94.8% of the patients underwent tumor resection, and total resection was achieved in 51.1%. Radiation therapy after surgery was also adopted for 42.3% of the patients. In this cohort, 478 patients survived, and 172 patients died, with only 6.3% of the patients having died due to primary cSFTs. The median survival time was 57 months.

**Table 1:** The demographic characteristics, treatment, and follow-up of these patients with CNS solitary fibrous tumors.

Variable	Number	Percentage (%)
Age at diagnosis (years)		
Mean ± SD	52.73 ± 16.815	
Median	55	
<60	410	63.1
≥60	240	36.9
Survival month		
Mean ± SD	62.95 ± 46.851	
Median	57	
Sex		
Female	322	49.5
Male	328	50.5
Race and origin		
Hispanic (All Races)	120	18.5
Non-Hispanic	530	81.5
Tumor location		
Brain	524	80.6
Spine	87	13.4
Not otherwise specified (NOS)	39	6
Phenotype		
Haemangiopericytoma phenotype	523	80.5
Solitary fibrous tumour phenotype	127	19.5
Behavior code ICD-O-3		
Benign	156	24
Borderline malignancy	273	42.0
Malignant	221	34.0
Tumor size		
≤3cm	110	16.9
>3cm	400	61.5
Unknown	140	21.5
Distant metastasis		
No	557	85.7
Yes	20	3.1
Unknown	73	11.2
Surgery		
No surgery	34	5.2
Partial resection	284	43.7
Total resection	332	51.1
Radiation after surgery		
No radiation and/or cancer-directed surgery	375	57.7
Radiation after surgery	275	42.3
Follow-up		
Alive	478	73.5
Dead (attributable to this tumor)	41	6.3
Dead (attributable to other cause)	131	20.2

**Figure 3:**
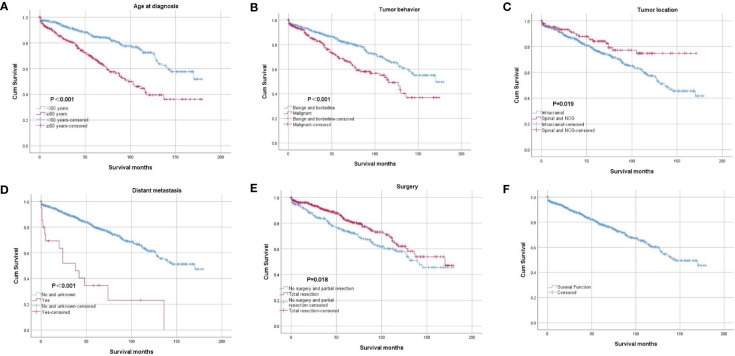
Kaplan–Meier curve of overall survival. The patients were stratified by age at diagnosis **(A)**, tumor behavior **(B)**, tumor location **(C)**, distant metastasis **(D)**, and surgery **(E)**. Picture **(F)** showed the Kaplan–Meier curve of the whole cohort.

The results of binary logistic regression analysis showed that intracranial tumors and tumors with distant metastasis tended to be malignant (**Table 2**). The OS rates at 5 and 10 years after diagnosis were 78.3% and 60.2%, respectively (**Figure 3**). The results of the Kaplan–Meier curves and log-rank test showed that patients older than 60 years of age who were diagnosed with intracranial tumors, malignant tumors and distant metastasis had worse OS outcomes than their counterparts (**Figure 3**). The results also showed that the patients undergoing total resection had better OS, but the effect of radiation therapy after surgery was not significant. The results of multivariable Cox proportional hazard regression analysis showed that patients older than 60 years of age who were diagnosed with intracranial tumors, malignant tumors and distant metastasis had worse OS outcomes than their counterparts (**Table 3**).

**Table 2:** Results of binary logistics regression.

Variables	OR(95%CI)	P value
Age at diagnosis (years)		
<60	Reference	
≥60	1.032 (0.724-1.471)	0.862
Sex		
Female	Reference	
Male	0.838 (0.597-1.175)	0.305
Race		
Non-Hispanic	1.329 (0.842-2.099)	0.222
Hispanic	Reference	
Tumor location		
Intracranial	Reference	
Spinal cord and NOS	0.579 (0.358-0.937)	0.026
Tumor size		
≤3 cm and Unknown	Reference	
>3 cm	1.313 (0.908-1.9)	0.148
Distant metastasis		
No and unknown	Reference	
Yes	51.126 (6.68-391.298)	<0.001

**Table 3:** Results of multivariable Cox regression.

Variables	Multivariable Cox regression	
	HR(95%CI)	P value
Age at diagnosis (years)		
<60	Reference	
≥60	2.473(1.811-3.377)	<0.001
Sex		
Female	Reference	
Male	1.153(0.847-1.57)	0.365
Race		
Non-Hispanic	0.938(0.626-1.407)	0.758
Hispanic	Reference	
Tumor location		
Intracranial	Reference	
Spinal cord and NOS	0.545 (0.337-0.883)	0.014
Tumor size		
≤3 cm and Unknown	Reference	
>3 cm	0.891 (0.646-1.231)	0.485
Distant metastasis		
No and unknown	Reference	
Yes	3.555 (1.94-6.516)	<0.001
Tumor behavior		
Benign and borderline	Reference	
Malignant	1.677 (1.194-2.354)	0.003
Surgery		
No surgery and Partial resection	Reference	
Total resection	0.809 (0.592-1.104)	0.182
Radiation after surgey		
Yes	0.769 (0.554-1.067)	0.116
No	Reference	

## Discussion

SFTs account for < 1% of all primary CNS tumors and most commonly affect adults in the fourth to sixth decades of life (1). The tumors are typically Dural-based and often supratentorial, with approximately 10% being spinal (1). It has been reported to affect males more frequently than females (13–15). Previous reports (4, 13–15) have revealed that cSFT has a higher rate of extracranial metastases, especially in bone, the lung, and the liver. In clinical practice, many cases cannot get information on distant metastasis just because they are incredibly ill. Furthermore, the information on extracranial metastases was unknown for 11.2% of cases in our cohort. Our study’s rate of distant metastases may not explain the actual situation. Most of cSFTs are WHO grade II (4, 15), and the rate of recurrence for the hemangiopericytoma phenotype was reported to be > 75% at 10 years (1). Pathology and immunohistochemistry are the standard diagnostic methods, but most cases of cSFT can be confirmed by characteristic imaging findings (16). In our cohort, most of the clinical features of cSFT were consistent with previous reports, such as the age at diagnosis, tumor location, tumor size, WHO grade and so on. However, we found that the rate of distant metastases was 3.1%, which is much lower than that in a previous report. We also did not find a significant difference in the distribution of patients by sex, with the female:male ratio being almost one. Because the SEER database does not collect information on imaging features, we could not analyze the imaging characteristics of cSFT. The hemangiopericytoma phenotype accounted for 80.5% of the cases in this cohort. Due to the recent recognition of cSFT as a single entity (7), we did not divide the patients into different groups by phenotype.

The results of the Kaplan–Meier curves showed that older patients, patients with intracranial cSFTs, malignant tumors, and distant metastasis had worse OS outcomes than their counterparts. Older patients are more vulnerable to suffering from other diseases, and they generally get worse OS than younger patients. Furthermore, it is apparent to understand that patients with malignant tumors get worse OS than patients with benign tumors. Why do patients with intracranial cSFTs have worse OS than those with spinal cSFTS? This could be caused by an increased morbimortality for patients with intracranial tumors. Moreover, we also found that intracranial cSFTs are more likely to be malignant than spinal cSFTs.

Because of the aggressive nature of SFTs, in addition to the first-line treatment choice of total resection, radiation therapy after surgery has been adopted by an increasing number of patients. However, the effect of radiation therapy remains controversial (17, 18). Rutkowski et al. conducted a systematic review of intracranial hemangiopericytoma, collecting the clinical data of 563 patients with intracranial hemangiopericytoma, and the results of their survival analysis showed that gross-total resection provides the greatest survival advantage and that the addition of postoperative adjuvant radiation does not seem to confer a survival benefit (13). Another systematic review of 523 patients with CNS hemangiopericytoma concluded that complete resection followed by adjuvant radiation improves survival (19). The results of a recent single-center retrospective analysis also showed that postoperative radiotherapy did not improve overall survival (17, 20). The results of the log-rank test and Cox regression in our study also confirmed that radiation therapy after surgery did not significantly improve OS in our cohort. Due to the aggressive nature of SFT, we still believe that radiation therapy should be considered, especially for patients in whom total resection cannot be achieved. A more detailed study should be conducted to further evaluate the effect of radiation therapy.

All cancer registrars in the United States began to collect information on benign and borderline tumors of the CNS in 2004 (21). We therefore set the research period as 2004-2018 to avoid the collection of incomplete data. The results of binary logistic regression analysis in our study showed that intracranial tumors and tumors with distant metastasis tended to be malignant. We also adopted binary logistic regression analysis to predict what kind of tumor tends to metastasize to other organs. However, only 3.1% of the tumors had distant metastasis, a rate far lower than that reported in previous studies (1, 4, 19). Because the percentage of positive results was too low to ensure the accuracy of the regression analysis, we adopted a descriptive method to analyze the prevalence of SFT patients with distant metastasis. At the same time, we wanted to include cancer-specific survival in the regression and survival analyses. Because the rate of death attributed to this CNS tumor was 6.3%, which is much lower than 15%, we chose to investigate overall survival instead to ensure the accuracy of the analysis. However, our study has several limitations, such as a lack of detailed clinical data, detailed treatment options, information on recurrence and geographic distribution of patients. Nevertheless, to the best of our knowledge, this is the largest cohort study on cSFT to date. We even included more cases than the recent systematic review published in 2021 (14). We believe that our study can help clinicians gain a better understanding of cSFT and make more suitable treatment decisions.

## Conclusion

We conducted a population-based analysis of patients with central nervous system SFTs. We found that cSFTs are rare, and most of them are WHO grade II or III. Intracranial cSFTs and cSFTs with distant metastasis tend to be malignant. Although tumor resection is the first choice of treatment for these patients, a more detailed study should be conducted in the future to further evaluate the effect of radiation therapy. In conclusion, patients older than 60 years of age who are diagnosed with intracranial tumors, malignant tumors and distant metastasis were found to have worse OS outcomes than their counterparts.

## Data availability statement

The raw data supporting the conclusions of this article will be made available by the authors, without undue reservation.

## Ethics statement

Ethical review and approval was not required for the study on human participants in accordance with the local legislation and institutional requirements. Written informed consent from the participants' legal guardian/next of kin was not required to participate in this study in accordance with the national legislation and the institutional requirements.

## Author contributions

TL, HX and YW drafted the manuscript. XD, ZJ and YW acquired the data and conducted the analysis. YW designed the study. All authors contributed to the article and approved the submitted version.
